# High effective coverage of vector control interventions in children after achieving low malaria transmission in Zanzibar, Tanzania

**DOI:** 10.1186/1475-2875-12-38

**Published:** 2013-01-29

**Authors:** Netta Beer, Abdullah S Ali, Delér Shakely, Kristina Elfving, Abdul-Wahiyd H Al-Mafazy, Mwinyi Msellem, Max Petzold, Anders Björkman, Karin Källander

**Affiliations:** 1 Department of Public Health Sciences, Karolinska Institutet, 171 77, Stockholm, Sweden; 2 Malaria Research Group, Infectious Disease Unit, Department of Medicine, Solna, Karolinska Institutet, Stockholm, Sweden; 3 Zanzibar Malaria Control Programme (ZMCP), Ministry of Health, Zanzibar, Tanzania; 4 Department of Medicine, Kungälv Hospital, Kungälv, Sweden; 5 Department of Infectious Diseases, Institute of Biomedicine, University of Gothenburg, Gothenburg, Sweden; 6 Akademistatistik-Centre for Applied Biostatistics, Sahlgenska Academy, University of Gothenburg, Gothenburg, Sweden; 7 Makerere University School of Public Health, Kampala, Uganda; 8 Malaria Consortium Africa, Kampala, Uganda

## Abstract

**Background:**

Formerly a high malaria transmission area, Zanzibar is now targeting malaria elimination. A major challenge is to avoid resurgence of malaria, the success of which includes maintaining high effective coverage of vector control interventions such as bed nets and indoor residual spraying (IRS). In this study, caretakers' continued use of preventive measures for their children is evaluated, following a sharp reduction in malaria transmission.

**Methods:**

A cross-sectional community-based survey was conducted in June 2009 in North A and Micheweni districts in Zanzibar. Households were randomly selected using two-stage cluster sampling. Interviews were conducted with 560 caretakers of under-five-year old children, who were asked about perceptions on the malaria situation, vector control, household assets, and intention for continued use of vector control as malaria burden further decreases.

**Results:**

Effective coverage of vector control interventions for under-five children remains high, although most caretakers (65%; 363/560) did not perceive malaria as presently being a major health issue. Seventy percent (447/643) of the under-five children slept under a long-lasting insecticidal net (LLIN) and 94% (607/643) were living in houses targeted with IRS. In total, 98% (628/643) of the children were covered by at least one of the vector control interventions. Seasonal bed-net use for children was reported by 25% (125/508) of caretakers of children who used bed nets. A high proportion of caretakers (95%; 500/524) stated that they intended to continue using preventive measures for their under-five children as malaria burden further reduces. Malaria risk perceptions and different perceptions of vector control were not found to be significantly associated with LLIN effective coverage.

**Conclusions:**

While the majority of caretakers felt that malaria had been reduced in Zanzibar, effective coverage of vector control interventions remained high. Caretakers appreciated the interventions and recognized the value of sustaining their use. Thus, sustaining high effective coverage of vector control interventions, which is crucial for reaching malaria elimination in Zanzibar, can be achieved by maintaining effective delivery of these interventions.

## Background

Recent success in malaria control in sub-Saharan Africa (SSA) has led to the renewed interest in malaria elimination and eradication [1,2]. In high-transmission areas, elimination may not be possible with currently available tools, but such tools can greatly reduce malaria transmission [1], as was demonstrated in Zanzibar [3,4] after wide-scale deployment of artemisinin-based combination therapy (ACT) and vector control interventions, ie, insecticide-treated nets (ITNs) and indoor residual spraying (IRS). Being a formerly high transmission area, Zanzibar is now targeting malaria elimination. A major challenge for Zanzibar is therefore to avoid resurgence of malaria, and this can only be done by maintaining high effective coverage of vector control interventions and comprehensive malaria case surveillance that would ensure quick response to potentially emerging epidemics [5].

In the late 1990s, the World Health Organization (WHO) started recommending and endorsing ITNs as the leading malaria prevention intervention. Despite the worry that the high ITN efficacy in controlled settings would not be translated into effectiveness under routine conditions, Lim *et al*. [6] show that routine scale-ups in several SSA countries resulted in 23% reduction in child mortality associated with ITN ownership, which is in line with the 18% seen in randomized control trials [7]. These findings suggest that the efficacious ITNs are well implemented to yield a high effective coverage. Effective coverage is an outcome that assesses how well an intervention had been implemented. It is defined as the proportion of the population in need of an intervention who are using an effective intervention [8], and is heavily affected by access and adherence to the intervention. While access to bed nets largely depends on delivery strategies [9-12], adherence to bed nets may be affected by various perceptions and beliefs [13-15].

In 2006, the WHO also started recommending the scale-up of IRS, in addition to ITNs [16]. Historically, IRS had been successful in eliminating malaria from areas with unstable transmission in the 1940s-60s. Marked, although temporary, malaria reduction was also documented in some areas in SSA, including Zanzibar [17]. In southern African countries, a significant decrease in malaria burden due to large scale and sustained application of IRS, was observed [18]. Despite its success in malaria reduction, there is general lack of evidence on the health impact of IRS from formal trials, especially in stable malaria settings [19]. Unlike effective coverage of ITNs, which also depends on continuous adherence by community members, IRS only requires community members to allow spraying the house, and no further action is necessary. Although ITNs seem to be more effective than IRS in areas with high endemicity [20-22], both interventions are often done simultaneously. In some cases, combining IRS and ITN was shown to have an additive effect [23,24]. Additive effects, however, are expected to vary with insecticides used, coverage and vector characteristics [25].

While effective coverage of both ITNs and IRS has previously been high in Zanzibar, there was a fear that a perceived lowered risk in the community may impede adherence to the vector control interventions as the malaria burden declines. Reduction in perceived susceptibility has previously been shown to lower adherence to preventive measures; one example is vaccination for childhood illnesses where vaccines are becoming less appreciated for their benefits, and instead more attention is given to their side-effects after the incidence of illnesses is reduced [26,27]. In the malaria field, a lower perceived risk of malaria in the dry seasons has been identified, and was suggested as one of the reasons for seasonal fluctuation in bed-net use, along with lower perceived nuisance from mosquitoes and the discomfort of sleeping under a bed net due to heat and humidity [15]. Although reduction in bed-net use during the dry seasons was previously documented [28,29], there is no evidence of reduced bed-net use due to overall reduction in malaria burden. The aim of this study was to assess effective coverage of malaria preventive measures following the significant reduction in malaria burden.

## Methods

### Study area

The study was conducted during June-July 2009 in two districts of Zanzibar: North A district (on Unguja island) and Micheweni district (on Pemba island). Zanzibar is an archipelago off the coast of mainland Tanzania. It previously had high and stable transmission of *Plasmodium falciparum* malaria, with malaria burden peaking during the rainy seasons; the long or heavy rains (Masika) between March/April and May/June and the short rains (Vuli) from October to December. However, in recent years there has been a dramatic decrease in malaria prevalence as a result of implementation and reinforcement of different malaria control measures [3,4].

In May 2005, the overall ITN use in children under five in Zanzibar was documented at 40%, with the Micheweni district having the lowest under-five ITN use (<10%) [30]. Consequently, retreatment campaigns were carried out in Micheweni during 2005, followed by a targeted mass distribution of long-lasting insecticidal nets (LLINs) in August 2005. The distribution campaign was scaled-up to other districts in early 2006. The LLINs distributed were blue rectangular Olyset® nets that were free of charge to all children under five and pregnant women. Free mass distribution of LLINs also took place from 2008 till 2009. In this distribution all households received two LLINs, except for households with a single resident that received only one net. The majority of LLINs were blue rectangular Olyset® nets, but some were white LLINs that cannot be easily distinguished from conventional nets.

From 2006 till 2009, there have been four rounds of IRS, targeting all households in Zanzibar (excluding Stone Town), with the last one implemented approximately six months before the survey was conducted. The insecticide used for IRS is the synthetic pyrethroid lambda-cyhalothrin (ICON).

### Sampling and sample size

A cross-sectional household survey using two-stage cluster sampling technique [31] was used. The selected sampling units, the *shehias* (the smallest administrative unit which comprises several villages), were the same as those randomly selected in a previous survey in 2006 [8]. However, the 22 shehias chosen in 2006 had been restructured by the Zanzibar authorities into 32 shehias due to population growth; hence 32 sampling units were used in this survey. Households were randomly selected from these sampling units in proportion to shehia size. Assuming a proportion of 50% of under-five children sleeping under bed nets, and accounting for a cluster effect of two, a sample size of 192 under-five children was needed to determine LLIN use with an absolute precision of ±10% and a 95% confidence interval. Since the survey was done in conjunction with the annual malaria cross-sectional survey conducted in the two districts, the total number of caretakers interviewed was larger than needed (560 in total), and some households had more than one under-five child. If a household on the sampling list could not be found, or consent could not be obtained, it was replaced by another household from a reserve list. Participation consent by community members was generally high.

### Data collection

Data were collected in North A and Micheweni districts right after the rainy season through household interviews with caretaker, preferably mothers, using a structured questionnaire with closed and open-ended questions in Kiswahili. Nineteen trained interviewers, who were all health professionals, received one week of training on the interview technique. The caretakers were interviewed in their homes, and asked about different perceptions and beliefs on the malaria situation in Zanzibar, vector control interventions (ie, bed nets and IRS), current vector control coverage, and intention to continue the use of vector control interventions in the event that malaria burden further decreases.

### Ethical considerations

The study was approved by the Zanzibar Medical Research Ethical Committee (ZAMEC). District leaders and local leaders (*shehas*) were informed about the study, and before starting the interviews, respondents signed an informed consent form.

### Data analysis

Data were single entered in CSPRO 4 by a data entry clerk, checked for consistency and errors, and analysed in STATA 10. Open-ended answers were coded into different categories and analysed quantitatively. Frequencies and proportions of perceptions and bed-net usage were computed. Factors associated with under-five LLIN use were identified using bivariate logistic regression. All variables with a *p*-value ≤0.25 in the bivariate analysis were included in the multiple logistic regression model after they were checked for colinearity. *p*-*values* were adjusted for cluster effect on the shehia level using the STATA svy command.

The study population was grouped into socio-economic quintiles based on an asset index, which was created using principle component analysis (PCA) [32]. The index was based on type of floor, walls and roof, source of water and light, type of toilet and cooking facilities, and owning 20 different assets. LLIN and IRS effective coverage in under fives belonging to different socio-economic quintiles was compared.

## Results

A total of 560 caretakers of 693 under-five children were interviewed from 292 households in Micheweni and 268 households in North A. The majority of the respondents were mothers (62%). The caretakers had a mean age of 34 and varying levels of education (Table 1).

**Table 1:** **Characteristics of respondents** (**n** = **560**)

**Variable**	**Number (%)**
District	
Micheweni (MI)	292 (52%)
North A (NA)	268 (48%)
Respondent's sex	
Female	412 (74%)
Male	148 (26%)
Respondent's closest relationship to the children*	
Mother	347 (62%)
Father	108 (19%)
Grandmother	35 (6%)
Sibling	43 (8%)
Other	27 (5%)
Education level**	
No education	263 (47%)
Primary education	179 (32%)
Secondary education	90 (16%)
Informal education (Koran studies)	15 (3%)

* Some caretakers have multiple relationships with several children in the household.

** Missing information for 13 respondents.

Of the 660 under-five children for whom information on bed-net use was available, 85% (563/660) had slept under a bed net the previous night. The majority of the children had slept under an LLIN (70%; 459/660), whereas 7% (44/660) slept under a conventional treated net (ITN) and 9% (60/660) slept under an untreated net (Table 2). Seasonal bed-net use was identified, whereby 25% of the caretakers (125/508) reported that their under fives were using the bed nets seasonally. There was no statistically significant difference in LLIN usage between North A district (71%; 241/338) and Micheweni district (68% ;218/322) (p = 0.45). IRS coverage was greater than bed-net coverage; 92% (515/560) of the houses were reported to have been sprayed with IRS in the previous 12 months, resulting in effective IRS coverage of 95% of the children (638/675).

**Table 2:** **Effective coverage of under**-five children in Micheweni and North A districts^*^

	**North A**	**Micheweni**	**Total**
**LLINs**	**71%** (**241**/**338**)	**68%** (**218**/**322**)	**70%** (**459**/**660**)
Conventional treated nets	8% (26/338)	6% (18/322)	7% (44/660)
Conventional untreated nets	9% (31/338)	8% (25/322)	9% (60/660)
Total treated nets (LLINs and ITNs)	79% (267/338)	73% (236/322)	76% (503/660)
Total bed nets	89% (301/338)	81% (262/322)	85% (563/660)
**IRS**	**95%** (**324**/**342**)	**94%** (**314**/**333**)	**95%** (**638**/**675**)
**Both interventions** (**LLINs and IRS**)	**68%** (**225**/**329**)	**64%** (**201**/**314**)	**66%** (**426**/**643**)
**At least one of the interventions** (**LLINs or IRS**)	**98%** (**321**/**329**)	**98%** (**307**/**314**)	**98%** (**628**/**643**)

^*^ Slight changes in percentages (%) are due to rounding issues.

Of the 643 under-five children for which both IRS and LLIN effective coverage information was available, 66% (426/643) were covered by both interventions, 3% (21/643) were only covered by bed nets, and 28% (181/643) were only covered by IRS. This means that merely 2% (15/643) of the under-five children were not covered by any one of the interventions (Table 2).

**Figure 1:**
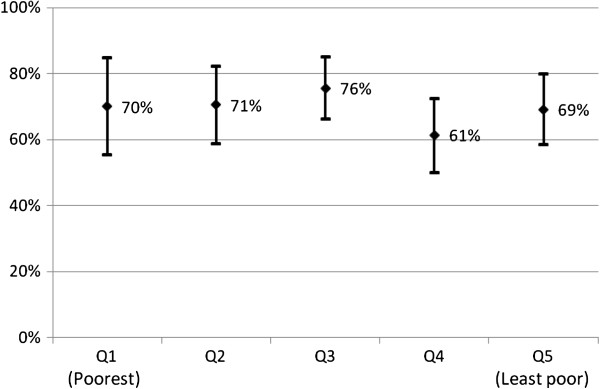
**Proportion of children under five years old from different socio**-**economic quintiles sleeping under LLINs, with 95% CI.**

**Figure 2:**
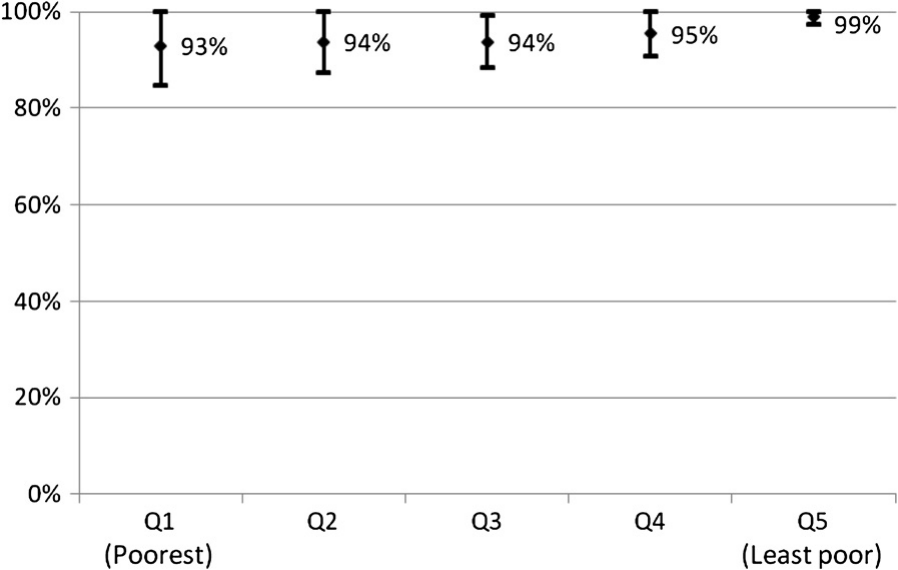
**Proportion of children under five years old from different socio**-**economic quintiles living in a house that was sprayed with IRS, with 95% CI.**

Effective coverage of LLINs was similar in the poorest income group (70%) and the least poor (69%) (Figure 1), whereas IRS effective coverage tended to be higher in the least poor (99%) compared with the poorest (93%), but the difference was not statistically significant (p = 0.055) (Figure 2).

The majority (60%; 335/557) of caretakers stated that the general current health situation in Zanzibar was better than it was five years ago. When asked specifically about the malaria situation, improvement was mentioned by 81% (452/560) and 87% (484/559) felt that the malaria burden had been reduced. While a majority (78%; 432/556) of caretakers believed that malaria was a serious health issue five years ago, many (66%; 363/552) no longer saw it as a serious health problem. Despite the perceived risk reduction, children were still viewed as more vulnerable to malaria than adults and 83% (467/560) of caretakers perceived children to be the age group most at risk of contracting malaria. Children were also considered to be the group with the highest risk of developing malaria complications, as stated by 80% (450/560) of caretakers. Risk perceptions, however, were not found to be significantly associated with effective coverage of LLINs in children under five.

According to 41% (231/560) of the caretakers, one of the reasons for malaria reduction in Zanzibar was the use of bed nets, whereas 37% (206/560) mentioned the use of IRS. Other reasons mentioned included availability of malaria medication (10%; 55/560) and environmental cleanliness (5%; 30/560). Seventy-four percent (414/560) stated that malaria is best prevented by the use and treatment of bed nets. Environmental cleanliness was the second most frequently stated malaria preventive measure, mentioned by 30% (167/560) of caretakers, while IRS was only mentioned by 18% (100/560).

The vast majority (96%; 535/556) of caretakers agreed to the statement that bed nets were useful in preventing malaria, and 98% (547/556) agreed that nets were useful in preventing mosquito bites. The majority (89%; 495/556) also agreed that IRS can be useful in preventing malaria and 88% (485/554) thought that it was useful in preventing mosquito bites. Mosquito reduction was the most prominent (48%; 269/560) spontaneously stated advantage of IRS, with 71 (13%) specifically mentioning malaria mosquitoes. Insect reduction, especially of cockroaches and bed bugs was mentioned by 41% (230/560), while malaria reduction was mentioned by only 19% (104/560). Side-effects of IRS, such as itching and increased mosquito and insect populations (especially of bed bugs), were mentioned by 20% (112/550).

The importance of sustaining malaria interventions after malaria burden further decreases was affirmed by 95% (500/524) of the caretakers who mentioned that they would continue using preventive measures for their under-five children; 93% (502/537) of the caretakers acknowledged the importance of continuing bed net use. Of caretakers who spontaneously mentioned reasons for continued bed net use, mosquito prevention was specifically mentioned by 50% (234/472) and malaria protection was mentioned by 27% (128/472). Other reasons included insect prevention (16 caretakers), continuing the habit (12 caretakers) and protection against the cold (three caretakers). Sustained use of IRS after malaria reduction was affirmed by 89% (487/546) of the caretakers, mainly for mosquito prevention, mentioned by 37% (155/424) of those who gave a reason for continued IRS. "Prevention is better than cure" was also stated by a few caretakers as the reason for continued use of these interventions.

In bivariate analysis, caretakers thinking malaria is currently not a serious health issue (OR = 1.41, p = 0.026) was associated with an under-five child sleeping under an LLIN. In multivariate analysis, which also included spontaneously mentioning bed nets as a way of preventing malaria, intending to continue prevention methods for under-five children and thinking it is useful to combine several preventive measures together (*p*-value ≤0.25), none of the perception variables remained significantly associated with LLIN usage.

Most caretakers (85%, 456/536) found it useful to combine several preventive measures together, and over 20% (114/555) of the caretakers reported using other means of malaria prevention in addition to bed nets and IRS. The most widely reported additional prevention measure was environmental cleanliness, canned insecticide, insecticide coils and other devices. Other prevention measures motioned included physical barriers against mosquitoes and actions against mosquito breeding.

## Discussion

The overall effective coverage of vector control interventions in children under five was found to be extremely high, at 98%, mostly due to high IRS coverage. Maintaining such high coverage is a key driver for keeping the malaria prevalence low and reducing the risk of malaria resurgence. Thus, it improves the prospect of malaria elimination in Zanzibar [5].

Seventy percent of the under-five children were sleeping under an LLIN. Effective coverage was equitable, ie, equally high in the poorest compared to the least poor socio-economic group. Targeted free mass distribution campaigns were previously found to result in high and equitable under-five coverage in Zanzibar [8]. In the current study, effective ITN coverage among under-fives was found to be 76% after an untargeted mass distribution. This figure is comparable to the effective ITN coverage of 73% in Sierra Leone and 62% in Nigeria [33,34], following a similar distribution strategy.

IRS effective coverage was much higher; covering 95% of the under-five population, and although it was lower in the poorest compared to the least poor quintile, the difference was not statistically significant. This is also likely due to the fact that IRS is delivered free of charge to all households.

The majority of children (66%) were protected by both LLINs and IRS. Although simultaneous use of LLINs and IRS could benefit from an additive effect, especially if different insecticides are used [25], Zanzibar had chosen to use pyrethroids for both LLINs and IRS. Nevertheless, the implementation of these two interventions simultaneously elevated the effective coverage by at least one malaria prevention intervention for under-five children.

Given the relatively lower effective coverage of LLINs and the fact that high seasonal usage pattern was detected in the study, as was observed in other studies [13,28,29], it will be important to continue IRS efforts until LLIN coverage can be further improved.

Malaria reduction in Zanzibar is well established, as shown by cross-sectional surveys and health facility records [3,4]. This study indicates that caretakers have noted this reduction, and that low malaria risk perceptions were not found to negatively influence LLIN use, as was previously indicated in qualitative studies in Vanuatu [35] and Zanzibar [36].

Bed nets were highly appreciated by caretakers, and were the most commonly spontaneously mentioned method of preventing malaria. In addition to being useful in preventing malaria, they were also perceived as useful in preventing mosquito bites. This added benefit of bed nets as a way of preventing mosquito nuisance has been documented previously [35-38]. However, in this study, perceptions about bed nets were not found to be significantly associated with LLIN effective coverage.

While coverage of IRS was higher than that of bed nets, it was slightly less appreciated for reducing malaria and mosquitoes. This is in line with studies in Mozambique which have shown that acceptance of IRS relied more on sociopolitical factors rather than perceived benefits of malaria and mosquito prevention [39]. Furthermore, one in five caretakers in this study mentioned that IRS also had disadvantages such as itching, which has also been documented previously [40]. Other side effects included insect increase (in particular in bed bugs), which has also been reported from studies where DDT was used for IRS [41].

This study shows that perceptions of malaria risk, as well as the perceived benefits of vector control interventions, be it malaria prevention or mosquito nuisance prevention, are not significantly associated with the effective coverage of these interventions. Most net distributions are usually accompanied by intensive health information campaigns that focus on improving knowledge and awareness to increase the use of nets against malaria. However, it remains unclear what factors do in fact influence community members to comply with and use these interventions. Factors that were not investigated in this study, and that were shown to influence acceptance of some interventions, include sociopolitical aspects, like those found to influence IRS acceptance in Mozambique [39]. More qualitative research to elucidate these aspects would be beneficial in designing more effective Behaviour Change Communication (BCC) campaigns to accompany these interventions.

While the majority of community members stated an intention to continue using malaria prevention methods as malaria further decreases, community members' intentions to continue adherence to bed nets and IRS will not result in high effective coverage unless high access to these interventions is maintained.

### Methodological considerations

Most questions in the structured questionnaire were dichotomous closed questions with "yes" or "no" answers. These included the questions on perceived risk of malaria, perceived usefulness of bed nets and IRS in preventing malaria and mosquito bites, perceived usefulness of combining preventive measures and continued use of preventive measures after malaria further decreases. Although giving a dichotomous answer to these qualitative-natured questions could cause a bias, it was deemed as an appropriate way to quantify these perceptions. Another option would have been to use other quantitative techniques, such as Likert-type scales, which could potentially have given more nuanced responses. However, following the dichotomous questions, open-ended questions were used, where the participants could further explain or justify their answers.

Use and willingness to use malaria prevention might have been overestimated due to desirability bias. This bias may have been increased due to the fact that the survey was identified with the Zanzibar Malaria Control Program and the interviewers were health professionals. However, an attempt to minimize this risk was made through emphasizing the importance of creating a comfortable environment during the interview.

## Conclusion

While the majority of caretakers felt that malaria had been reduced in Zanzibar, effective coverage of vector control interventions remained high. Caretakers appreciated the interventions and recognized the value of sustaining their use. Thus, sustaining high effective coverage of vector control interventions, which is crucial in reaching malaria elimination in Zanzibar, can be achieved by maintaining effective delivery of these interventions.

## Competing interests

The authors declare that they have no competing interests.

## Authors’ contributions

NB-conception and design of the study, design of study tools, data collection, data management, analysis and interpretation of data, drafting the paper, revising the paper. ASA-conception and design of the study, design of study tools, analysis and interpretation of data, drafting the paper, revising the paper. DS-conception and design of the study, design of study tools, revising the paper. KE-conception and design of the study, design of study tools, revising the paper AHA-design of study tools, data management, analysis and interpretation of data, drafting the paper. MM-design of study tools, data collection, revising the paper. MP-data analysis and interpretation of data, revising the paper. AB-conception and design of the study, design of study tools, analysis and interpretation of data, drafting the paper, revising the paper. KK-conception and design of the study, design of study tools, analysis and interpretation of data, drafting the paper, revising the paper. All authors read and approved the final manuscript.
